# Emerging trends and hotspots in metabolic dysfunction-associated fatty liver disease (MAFLD) research from 2012 to 2021: A bibliometric analysis

**DOI:** 10.3389/fendo.2023.1078149

**Published:** 2023-01-24

**Authors:** Yudi Liao, Liya Wang, Fang Liu, Yanyu Zhou, Xiaoqi Lin, Zijun Zhao, Saihong Xu, Dan Tang, Yingfu Jiao, Liqun Yang, Weifeng Yu, Po Gao

**Affiliations:** ^1^ Department of Anesthesiology, Renji Hospital, Shanghai Jiao Tong University School of Medicine, Shanghai, China; ^2^ NMPA Key Laboratory for Research and Evaluation of Narcotic and Psychotropic Drugs, Jiangsu Province Key Laboratory of Anesthesiology, Jiangsu Province Key Laboratory of Anesthesia and Analgesia, Xuzhou Medical University, Xuzhou, China; ^3^ Department of Gynecologic Oncology, International Peace Maternity and Child Health Hospital, Shanghai Jiao Tong University School of Medicine, Shanghai, China

**Keywords:** MAFLD, NAFLD, bibliometric analysis (BA), hotspots, CiteSpace

## Abstract

**Background:**

Metabolic dysfunction-associated fatty liver disease (MAFLD) has become the most common chronic liver disease. MAFLD is a major risk factor for end-stage liver disease including cirrhosis and primary liver cancer. The pathogenesis of MAFLD is complex and has not yet been clarified. To the best of our knowledge, few studies have conducted quantitative bibliometric analysis to evaluate published MAFLD research. In this study, we conducted a comprehensive analysis of MAFLD publications over the past decade to summarize the current research hotspots and predict future research directions in this field.

**Methods:**

Articles into MAFLD published from 2012 to 2021 were identified from the Science Citation Index-Expanded of Web of Science Core Collection. CiteSpace software, VOSviewer, the “bibliometrix” R package, and the Online Analysis Platform of Literature Metrology were used to analyze the current publication trends and hotspots.

**Results:**

We retrieved 13959 English articles about MAFLD published from 2012 to 2021. Primary sites of publication were dominated by the United States until 2014, when China became the source of most published MAFLD-related research papers. The United States was found to be the most engaged country in international cooperative efforts. Shanghai Jiao Tong University was the most productive institution. Loomba R was the most productive author with 123 articles. The co-cited keyword cluster tag showed ten main clusters: #0 liver fibrosis, #1 hemoglobin, #2 metabolic associated fatty liver disease, #3 egcg, #4 myocardial infarction, #5 heart disease, #6 pnpla3, #7 hepatocellular carcinoma, #8 noninvasive marker, and #9 children. Keyword burst analysis showed that gut microbiota was the highest-intensity research hotspot.

**Conclusion:**

In the past decade, the number of publications on MAFLD increased dramatically, especially in the last three years. Gut microbiota became an important research direction for etiological and therapeutic investigations into MAFLD. Insulin resistance was also a key factor in studying the development of MAFLD in recent years. Liver fibrosis was an important focus of disease development. This study provides systematic information, helps guide future research, and helps to identify mechanisms and new treatment methods for MAFLD.

## 1 Introduction

Metabolic dysfunction-associated fatty liver disease, formerly named non-alcoholic fatty liver disease (NAFLD), is a public health problem that is the most common chronic liver disease worldwide. The renaming from NAFLD to MAFLD in 2020 stemmed from a need for precise diagnosis and effective treatment (1). One-quarter of the global population is estimated to have MAFLD (2). MAFLD is already the fastest-growing cause of hepatocellular carcinoma (HCC) worldwide, especially in the USA, France, and the UK (3). In recent years, China surpassed the European countries and USA in terms of incidence (4). Worryingly, disease modeling suggests that the total number of individuals with MAFLD is expected to increase substantially to 314.58 million in China by 2030, representing the greatest increase in disease prevalence globally (5). MAFLD is a disorder characterized by excess accumulation of fat in hepatocytes. In up to 40% of individuals, there are additional findings of portal and lobular inflammation and hepatocyte injury [which characterize nonalcoholic steatohepatitis (NASH)] (6). Convincing evidence shows a strong association between MAFLD and the risk of developing multiple extrahepatic complications such as type 2 diabetes, cardiovascular disease (which is the main cause of mortality in people with MAFLD), chronic kidney disease, and some types of extrahepatic malignancies. The magnitude of this risk is related to the severity of MAFLD (especially the liver fibrosis stage). The latest models predict that the prevalence of MAFLD will continue to increase (7). It has been reported that bile acid signaling and metabolism and bile acid homeostasis are disrupted in patients with MAFLD, and that drugs targeting the farnesoid X receptor (FXR)-fibroblast growth factor 19 axis or bile acid conjugates might be beneficial in the treatment of NASH (8–10). Although steady progress has been made in improving our understanding of disease epidemiology, pathogenesis, and identifying therapeutic targets, the development of therapeutics has lagged behind these advances (11). The latest treatment-related studies have included modulation of gut microbiota and metabolites (12), hepatic microenvironment (for instance, adaptive and innate immune responses) (13), peroxisome proliferator-activated receptors (PPARs) (14), insulin resistance, impaired glycemic control, altered lipid metabolism (15), and others.

The large global prevalence of MAFLD has attracted major pressures on medical systems and imposed economic burdens, resulting in an increased demand for in-depth research in this field. However, in the past decade, few bibliometric articles have summarized the latest progress in this field and predicted research hotspots. The latest bibliometric study only summarized literature related to MAFLD research before 2014 (16). In this study, we summarize relevant MAFLD studies published from 2012 to 2021 and conduct a bibliometric analysis. Bibliometric analysis involves a timely and comprehensive review of publications during a specific period by analyzing their parameters such as the number of publications, authors, countries, and regions, references, keywords, etc. This approach provides a thorough overview of the intellectual landscape and helps researchers by highlighting the latest research trends (17–19). We aimed to analyze the publication trend of MAFLD from 2012 to 2021, to identify novel discoveries in pathogenesis and possible treatment measures, and to provide a panoramic overview of the research field for other researchers.

## 2 Materials and methods

### 2.1 Data sources and search strategies

**Figure 1:**
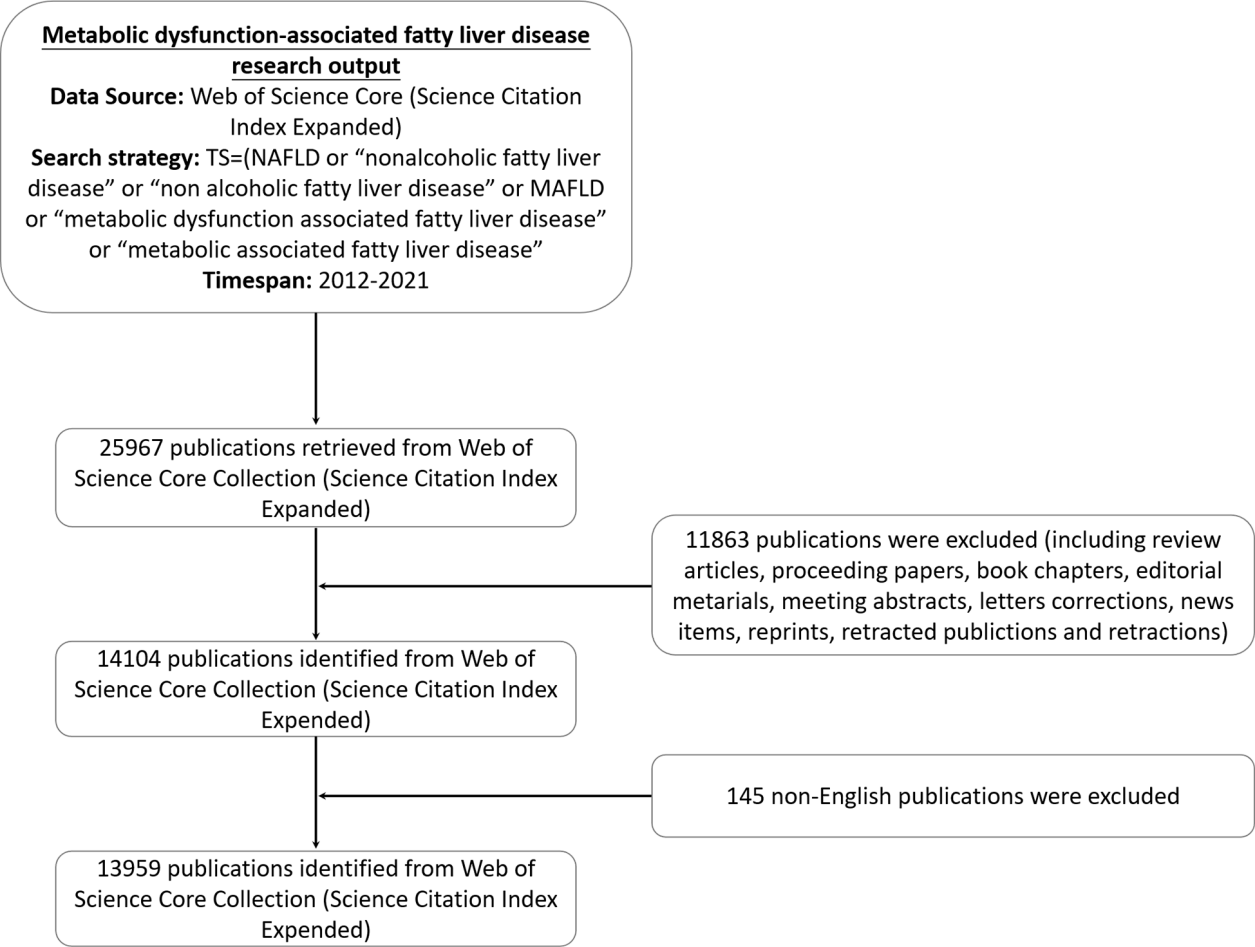
Flowchart for including and excluding publications.

We conducted a comprehensive literature search within the Science Citation Index-Expanded (SCI-E) of the Web of Science Core Collection (WoSCC) database for the period 2012-2021, on September 6, 2022. To reduce the deviation caused by frequent database updates, we completed the search and retrieval of data in one day. We retrieved relevant publications in WoSCC through the following search strategy: TS=(NAFLD or “nonalcoholic fatty liver disease” or “non alcoholic fatty liver disease” or MAFLD or “metabolic dysfunction-associated fatty liver disease” or “metabolic associated fatty liver disease”) from January 1, 2012, to December 31, 2021, and the “Document Type” was limited to “Articles” only. To confirm the accuracy of bibliometric analysis results, we examined all publications retrieved through the above search strategy by title, abstract, and year of publication. Exclusion criteria were as follows: (i) not related to MAFLD, (ii) non-article document types (such as comments, editorial materials, letters, and conference summaries), (iii) repeated publishing, and (iv) non-English language publications. We finally identified 13,959 articles on MAFLD research for econometric analysis. The detailed filtering is shown in **Figure 1**.

### 2.2 Bibliometric analysis

To describe all the literature features related to MAFLD studies, Web of Science (http://wcs.webofknowledge.com) was used to analyze retrieval results and extract the histogram showing the publication trends. We converted all data that met the requirements from WOSCC to TXT format and imported the data into CiteSpace analysis software (Drexel University, Philadelphia, PA, USA), bibliometric online platform analysis (http://biblimetric.com), VOSviewer 1.6.18 (Leiden University, Leiden, The Netherlands), and used the bibliometrix R package for visual processing.

Full records and references cited in these publications were downloaded from the WoSCC database, saved as a txt format, and imported into the CiteSpace software V6.1.3. The parameters of CiteSpace were as follows: time slicing (2012–2021), years per slice (1), term source (all selection), node type (choose one at a time), pruning (pathfinder), and visualization (cluster view-static, show merged network). CiteSpace was used to perform the bibliometric analysis. Visualization knowledge maps consist of nodes and links. Different nodes in a map represent elements such as a cited reference, institution, author, and country, and links between nodes represent relationships of collaboration/cooccurrence or co-citations. The publication number from the top 10 countries/regions and the top 10 most productive journals were exported from the Online Analysis Platform of Literature Metrology. The bibliometrix R package was used to output the high-frequency keywords as a world cloud map. Collaborations between countries/regions were analyzed by VOSviewer software.

## 3 Results

### 3.1 Quantity and trends analysis of published papers

**Figure 2:**
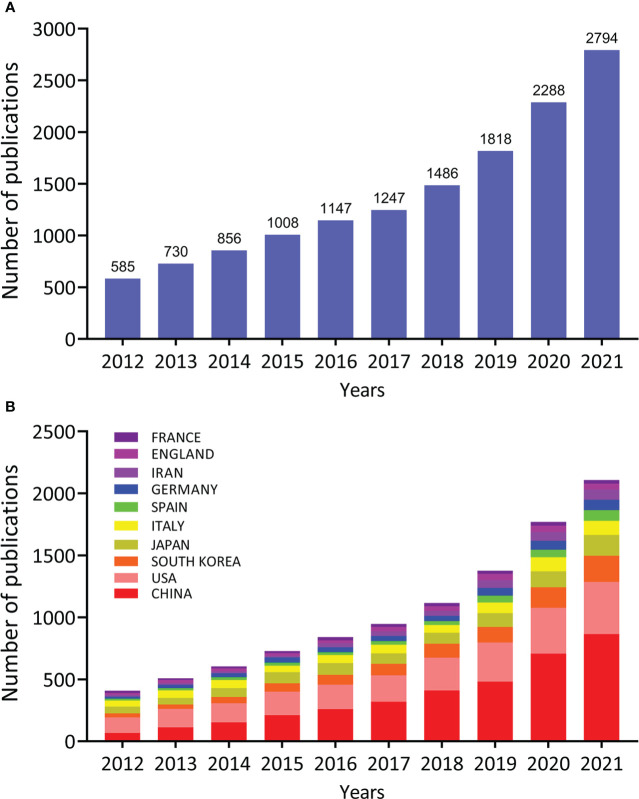
Quantity and trends analysis of published papers on the topic of MAFLD research between 2012 and 2021. **(A)** The number of annual research publications and growth trends on the topic of MAFLD from 2012 to 2021, export of results from Web of Sciences. **(B)** The number of annual research publications and growth trends on the topic of MAFLD from 2012 to 2021, export of results from the Online Analysis Platform of Literature Metrology.

In the SCI-E of WoSCC, the total number of papers published during 2012-2021 that met our inclusion criteria was 25,967. 11,863 articles were excluded due to improper article types (review articles, meta-analysis, litigation papers, or correction articles), and 145 were excluded because they were not published in English. According to the defined search criteria, 13,959 articles were extracted from WoSCC. As shown in **Figure 2A**, research into MAFLD can be approximately divided into two time periods. Publication in the early stage (2012–2017) occurred with moderate growth while the number of publications over the last four years (2018–2021) grew by nearly 1.5 times the former. The number of publications in 2021 was almost five times that of 2012, indicating that MAFLD has gradually increased in interest as a public health problem (20, 21) worthy of research efforts. Moreover, we used Microsoft Excel 2003 to build a growth trend model as follows: f(x)=4.1333x^3^-24979x^2^+5E+07x-3E+10 (R^2^ = 0.9988), which predicted that nearly 7000 articles will be published by 2025 (**Supplementary Figure 1**).

To figure out which countries or regions played a leading role in MAFLD research over the past 10 years, the number of articles published by different countries and regions was calculated by using the Online Analysis Platform of Bibliometrics (http://bibliometric.com/). The generated histogram shows the number of publications from the top 10 countries/regions over the last 10 years (**Figure 2B**). Notably, the United States and China have long dominated MAFLD research with China seeing the most rapid growth in publications over the past 10 years. The number of publications published by the United States is currently second after China.

### 3.2 Analysis of intercountry/region and inter-institutional cooperation

**Figure 3:**
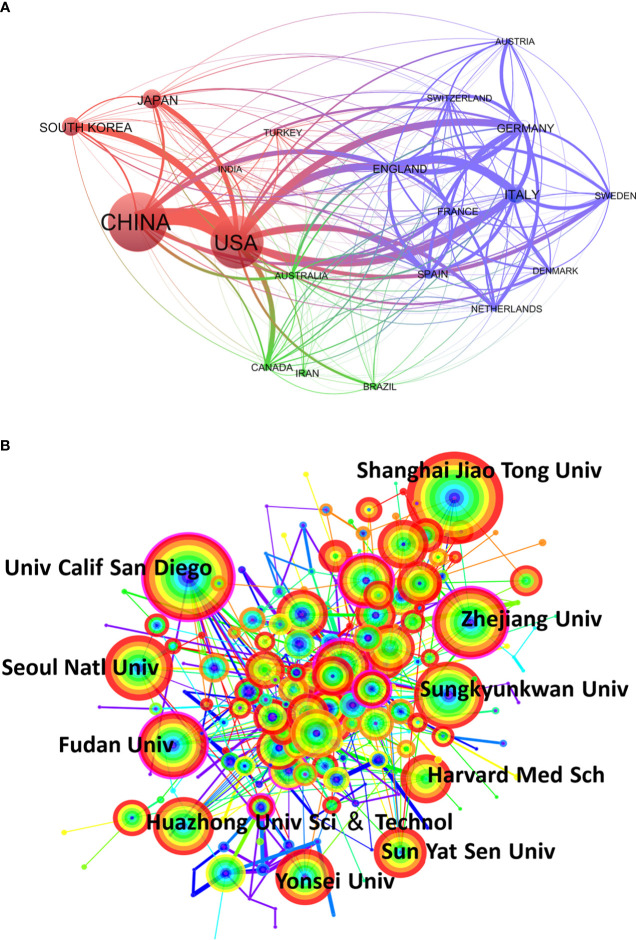
Network map of the collaboration analysis of MAFLD among countries/regions and institutions in 2012-2021. **(A)** Cooperative relationships between 118 countries/regions on the topic of MAFLD from 2012 to 2021. The image shows the top 20 counties/regions with frequent international cooperation, and the data was exported from **(B)** CiteSpace network map of institutions involved in MAFLD research. The top 10 most productive institutions are shown. The size of the concentric circle represents the number of articles published by each institution and the thickness of the connecting lines indicates the degree of cooperation between institutions. The redder the color of the concentric circle, the more productive the institution has been in MAFLD research in recent years.

We analyzed cooperative efforts among different countries using the VOSviewer (**Figure 3A**). The 13,959 articles were published by investigators from 118 countries and regions. **Figure 3A** shows the cooperative efforts of the top 20 countries. The figure represents the academic cooperation between countries/regions in the study of MAFLD, where each circle represents a country/region, and the link represents the strength of international cooperation. The size of the circle indicates the number of articles published, and the thickness of the connecting line indicates the degree of cooperation. We found that the United States tops international cooperation and China publishes the largest number of articles. We also found that the United States and China showed the closest cooperation.

To clarify the inter-institutional cooperation, we imported TXT files into CiteSpace software. CiteSpace analysis of institutions identified 193 nodes, indicating that 193 institutions participated in the 13,959 MAFLD publications included in the analysis (**Figure 3B**). The top 10 productive institutions are shown in the graph; each concentric circle represents one institution and the links indicate the strength of institutional cooperation. The size of the concentric circle represents the number of articles published by each institution and the thickness of the connecting lines indicated the degree of cooperation between institutions. More deep red colors of the concentric circles indicate greater productivity by the institution in MAFLD research in recent years. The research network presents a low-density map (density=0.0262), indicating that the research groups distributed across various institutions. The Shanghai Jiao Tong Univ had the largest number of published papers (338) and the most frequent collaborations with other institutions. Five of the top 10 institutions were from China and included the Shanghai Jiao Tong University (338), Zhejiang University (205), Fudan University (170), Huazhong University of Science & Technology (162), and the Sun Yat-Sen University (153). These are all famous higher education institutions in China, supported by research teams with strong scientific research capabilities. These findings suggest that Chinese institutions play a pivotal role in this field of research. The remaining 5 institutions with the largest number of publications were from the United States and South Korea, which have also made great contributions to MAFLD research. In the United States, the University of California San Diego (281) had the second-largest number of publications and Harvard Medical School (162) had the eighth-largest number of publications. In South Korea, the institutions included Seoul National University (198), Sungkyunkwan University (178), and Yonsei University (149).

### 3.3 Analysis of co-authorship networks and core author distribution

**Figure 4:**
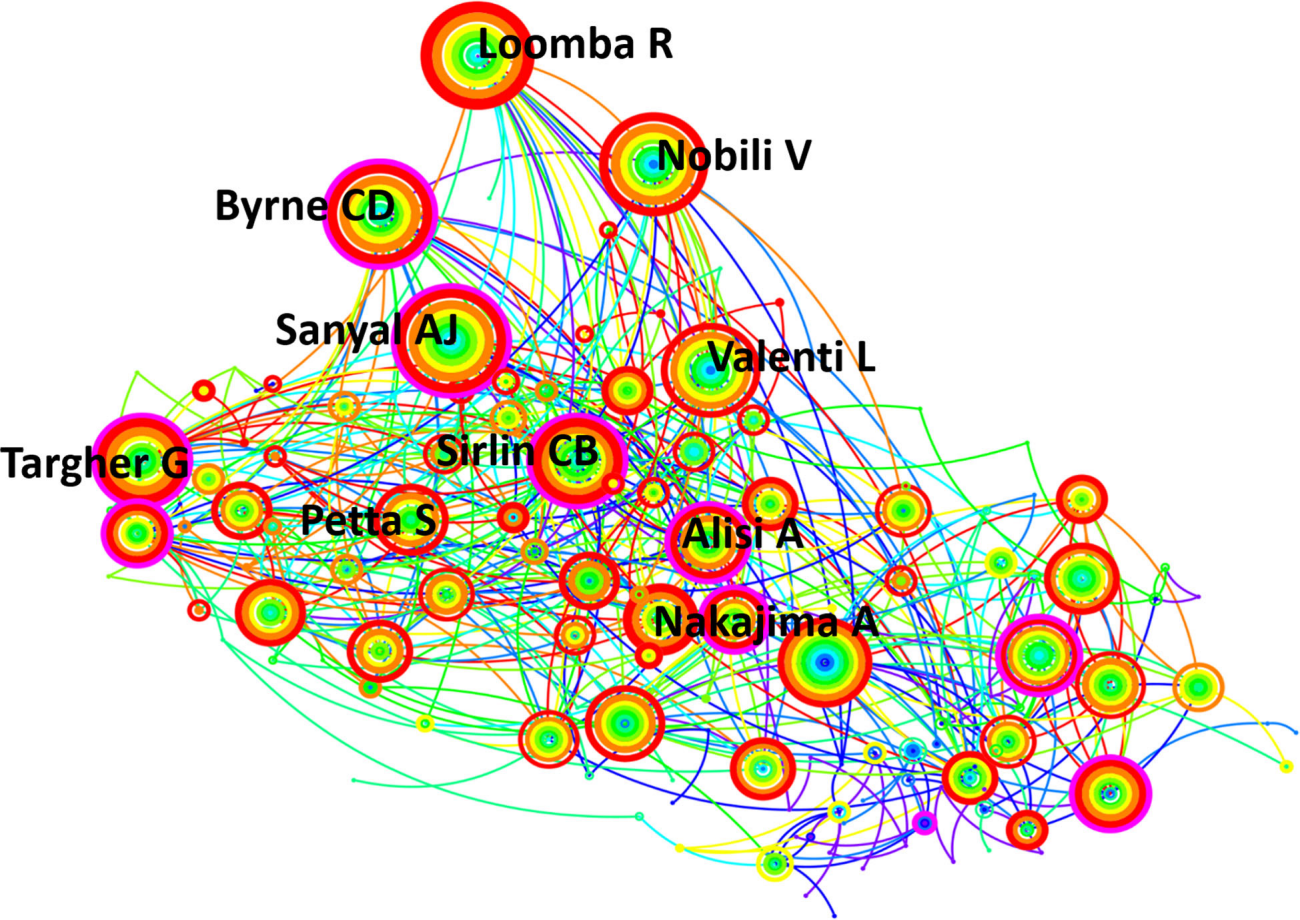
CiteSpace network of authorship in the field of MAFLD research. The top 10 authors with the most publications are shown. Each circle represents an author and lines between the circles represent the connections between authors.

In the past 10 years, 67,812 investigators participated in MAFLD research. Among them, 249 authors made the main contributions to the publication output (20 or more publications each). The top 10 most productive authors are shown in **Figure 4**. Each circle represents an author and the lines between the circles represent the connections between authors. Rohit Loomba from the NAFLD Research Center, Division of Gastroenterology, University of California at San Diego, La Jolla, CA, USA contributed the most articles (123), followed by Valerio Nobili from Hepato-Gastroenterology Disease Unit, Children Hospital IRCCS, Rome, Italy (82). From the cooperative network map of authors, the top 10 productive authors showed close collaborative relationships.

### 3.4 Analysis of journals

The 13,959 original articles were published in 1,696 academic journals. A bibliometrics online analysis platform was used to analyze journal influence. The top 10 most-cited journals are shown in **Table 1**, indicating that articles published in Hepatology were cited most frequently (11,517 times) during the past 10 years, followed by the *Journal of Hepatology* (7,376), *Gastroenterology* (4,926), *Plos One* (3,614), *World Journal of Gastroenterology* (3,326), *Liver International* (2,969), *Clinical Gastroenterology and Hepatology* (2,422), *Metabolism-Clinical and Experimental* (1,959), *Journal of Gastroenterology and Hepatology* (1,705), and *Gut* (1,635). Five of these journals are from the United States, while the others are from the Netherlands, China, Denmark, Australia, and England. Articles published in *Gastroenterology* had the highest average citation per paper (47.37).

**Table 1:** The top 10 most active journals that published articles in the MAFLD research from 2012 to 2021 (sorted by total citation).

Rank	Journal Title	Frequency	Total citations	Average citation per paper	Impact factor (2020)	Country	JCR
1	*Hepatology*	331	11517	34.79	17.298	USA	Q1
2	*Journal of Hepatology*	262	7376	28.15	30.083	Netherlands	Q1
3	*Gastroenterology*	104	4926	47.37	33.883	USA	Q1
4	*PLoS One*	465	3614	7.77	3.752	USA	Q2
5	*World Journal of Gastroenterology*	234	3326	14.21	5.374	China	Q2
6	*Liver International*	318	2969	9.34	8.754	Denmark	Q1
7	*Clinical Gastroenterology and Hepatology*	117	2422	20.70	13.576	USA	Q1
8	*Metabolism-Clinical and Experimental*	116	1959	16.89	13.934	USA	Q1
9	*Journal of Gastroenterology and Hepatology*	172	1705	9.91	4.369	Australia	Q2
10	*Gut*	65	1635	25.15	31.793	England	Q1

### 3.5 Analysis of document citations

We also sorted out the top 10 most-cited articles from the 13,959 identified articles. In **Table 2**, an article from *Nature* in 2012 ranked first in terms of citations (22). It proposes that the inflammasome mediates the development of MAFLD. The second article focuses more on the disease development and long-term outcomes of MAFLD (23). The third is a clinical trial published in the *Lancet*, which is a multicenter, randomized, placebo-controlled trial to study the therapeutic effects on MAFLD of the farnesoid X nuclear receptor ligand, obeticholic acid (24). Among the top 10 most cited articles, 3 were from *Gastroenterology*, 2 from *Hepatology*, 2 from *Journal of Hepatology*, and the rests were from *Nature*, *Lancet*, and *Nature Genetics*.

**Table 2:** The top 10 most cited articles from 13959 retrieved list in the MAFLD articles research from 2012 to 2021 (sorted by cited frequency).

Rank	Title	First Author	Journal	Year	Cited Frequency	DOI
1	Inflammasome-mediated dysbiosis regulates progression of NAFLD and obesity	Henao-Mejia	*Nature*	2012	1543	10.1038/nature10809
2	Liver Fibrosis, but No Other Histologic Features, Is Associated With Long-term Outcomes of Patients With Nonalcoholic Fatty Liver Disease	Angulo P	*Gastroenterology*	2015	1487	10.1053/j.gastro.2015.04.043
3	Farnesoid X nuclear receptor ligand obeticholic acid for non-cirrhotic, non-alcoholic steatohepatitis (FLINT): a multicentre, randomized, placebo-controlled trial	Brent A	*Lancet*	2015	1338	10.1016/s0140-6736 (14)61933-4
4	Fibrosis Stage Is the Strongest Predictor for Disease-Specific Mortality in NAFLD After Up to 33 Years of Follow-Up	Ekstedt M	*Hepatology*	2015	1169	10.1002/hep.27368
5	Weight Loss Through Lifestyle Modification Significantly Reduces Features of Nonalcoholic Stearohepatitis	Vilar-Gomez	*Gastroenterology*	2015	991	10.1053/j/gastro.2015.04.005
6	Exome-wide association study identifies a TM6SF2 variant that confers susceptibility to nonalcoholic fatty liver disease	Kozlitina J	*Nat Genet*	2014	684	10.1038/ng.2901
7	The severity of nonalcoholic fatty liver disease is associated with gut dysbiosis and shift in the metabolic function of the gut microbiota	Boursier J	*Hepatology*	2016	643	10.1002/hep.28356
8	Modeling NAFLD disease burden in China, France, Germany, Italy, Spain, United Kingdom, and United States for the period 2016-2030	Estes C	*J Hepatol*	2018	609	10.1016/j.jhep.2018.05.036
9	Evidence of NAFLD progression from steatosis to fibrosing-steatohepatitis using paired biopsies: Implications for prognosis and clinical management	Marchesini G	*J Hepatol*	2015	608	10.1016/j.jhep.2014/11.034
10	Elafibranor, an Agonist of the Peroxisome Proliferator-Activated Receptor-α and-δ, Induces Resolution of Nonalcoholic Steaohepatitis Without Fibrosis Worsening	Ratziu V	*Gastroenterology*	2016	603	10.1053/j.gastro.2016.01.038

**Figure 5:**
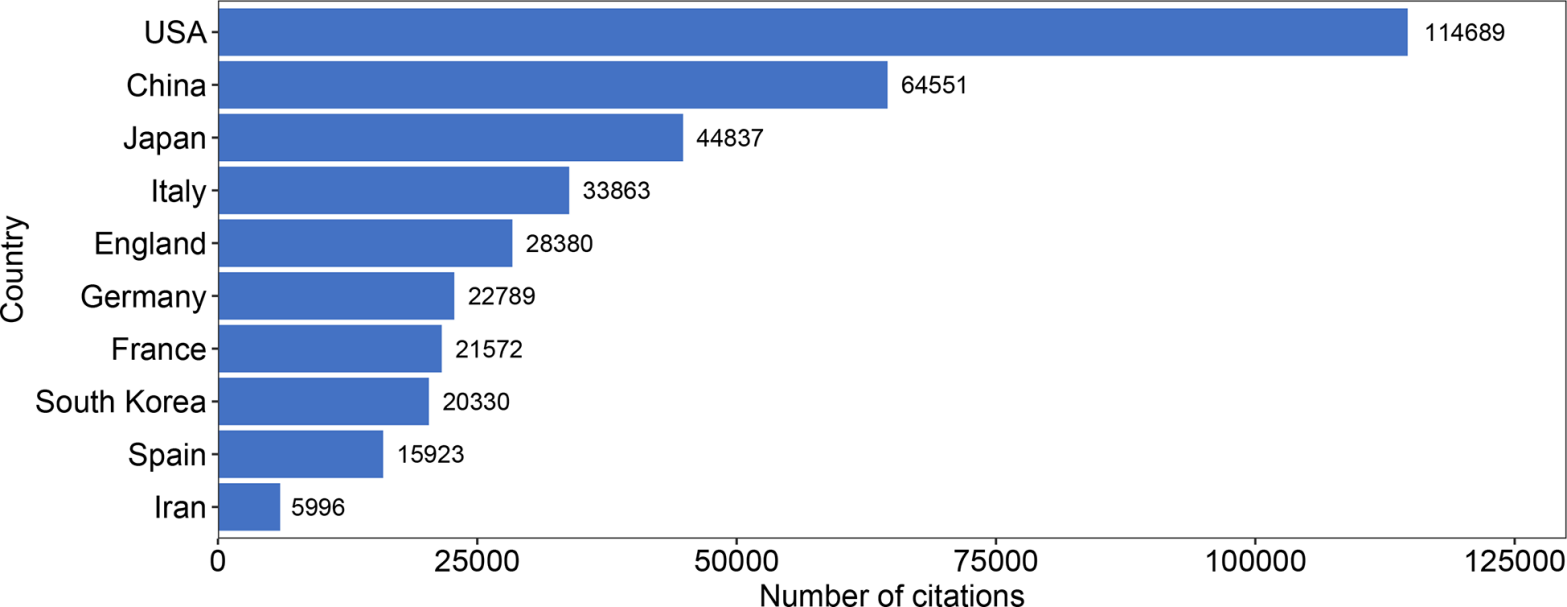
Bar graph of the total number of citations from 13959 retrieved articles among countries in 2012-2021. The top 10 countries with the highest total number of citations are shown.

In addition, we analyzed the total number of citations of all articles published by each country and presented the top ten countries according to the total number of citations in **Figure 5**. During this 10-year period, the total number of citations of articles published in the USA ranked first with 114689. China and Japan ranked second and third with 64551 and 44837. The 10^th^ place is Iran with a total of 5996 citations.

Combining the total number of publications and the total number of citations in each country, the average number of citations per article in each country can be obtained. The top ten countries sorted by average number of citations are shown in **Table 3**. The top three countries with the highest average number of citations per article were the France (average of 48.8 citations per article), England (37.3), and USA (33.7), and each article from these countries is cited more than 10 times on average. In terms of the 3402 articles from the USA, four articles were cited more than 1000 times, with the most cited being a paper by Jorge Henao-Mejia et al. with 1484 citations (22). The second most cited study in the USA was from Angulo P et al. with 1380 citations (23).

**Table 3:** The top 10 countries with the highest average number of citations per article from 13959 retrieved list in the MAFLD research from 2012 to 2021 (sorted by the average number of citations).

Rank	Country	Number of Publications	Total number of citations	Average number of citations
**1**	France	442	21572	48.8
**2**	England	760	28380	37.3
**3**	USA	3402	114689	33.7
**4**	Italy	1055	33863	32.1
**5**	Germany	777	22789	29.3
**6**	Spain	544	15923	29.3
**7**	South Korea	1064	20330	19.1
**8**	China	3862	64551	16.7
**9**	Iran	388	5996	15.5
**10**	Japan	3402	44837	13.2

### 3.6 Analysis of document co-citation and clustered networks

**Figure 6:**
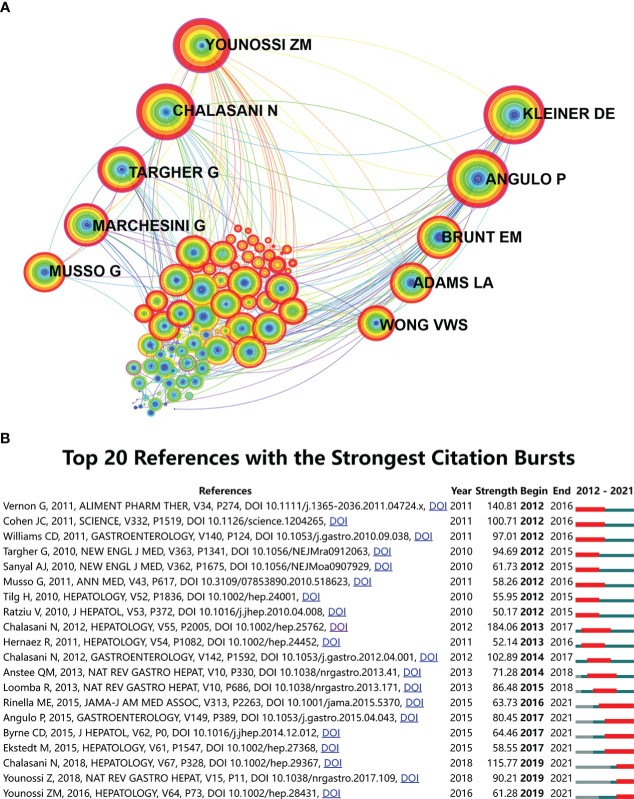
Reference co-citation network analysis of publications on the MAFLD research from 2012 to 2021. **(A)** CiteSpace co-citation map of 228,186 references on MAFLD, filter option showing the largest connected component only. Each node represents a reference and the link between nodes means that these articles were cited as references in the same article within the retrieved 13,959 articles. The size of the node is positively correlated with the frequency of citation and line thickness represents the correlation with the co-cited papers. The redder nodes represent that these papers are frequently cited in recent years while the more purple ones represent references cited in earlier years. The top 10 authors with the most cited articles are shown. **(B)** References with the strongest burst strength of the 13,959 citing articles on MAFLD research between 2012 and 2021. References marked in red indicate a sudden increase in the usage frequency of this reference during that period. Green represents a relatively unpopular period. The top 20 references with the strongest citation bursts are shown.

Document co-citation is a method to identify literature co-cited by a group of authors. This method is used to evaluate the relationship of two documents by visualizing their co-occurrence of citations. The 13,959 articles and their 228,186 references (excluding self-citations) retrieved from WoSCC were analyzed by CiteSpace to identify mutual homogeneity and then to cluster them. Map of co-citation references in CiteSpace on MAFLD research is presented in **Figure 6A**. The top 10 most cited authors are shown. Each node represents a reference and the link between nodes indicates that these articles were cited as references in the same article within the retrieved 13,959. The size of the node is positively related to the frequency of citation and line thickness represents the correlation with the co-cited papers. The red nodes represent more frequent citations in recent years while purple ones represent references cited in earlier years. The top ten articles sorted by cited frequency of citations are presented in **Table 4**.

**Table 4:** The top 10 high-cited references of 13959 retrieved articles in the MAFLD research from 2012 to 2021.

Rank	Title	First Author	Journal	Year	Cited Frequency	DOI
1	Global epidemiology of nonalcoholic fatty liver disease-Meta-analytic assessment of prevalence, incidence, and outcomes	Younossi ZM	*Hepatology*	2016	1726	10.1002/hep.28431
2	The diagnosis and management of nonalcoholic fatty liver disease: Practice guidance from the American Association for the Study of Liver Diseasea	Chalasani N	*Hepatology*	2018	960	10.1002/hep.29367
3	Global burden of NAFLD and NASH: trends, predictions, risk factors and prevention	Younossi ZM	*Nat Rev Gastroenrerol Hepatol*	2018	732	10.1038/nrgastro.2017.109
4	The diagnosis and management of non-alcoholic fatty liver disease: practice Guideline by the American Association for the Study of Liver Diseases, American College of Gastroenterology, and the American Gastroenterological Association	Chalasani N	*Hepatology*	2012	487	10.1002/hep.25762
5	EASL-EASD-EASO Clinical Practice Guidelines for the management of non-alcoholic fatty liver disease	EASL	*Diabetologia*	2016	454	10.1007/s00125-016-3902-y
6	Liver Fibrosis, but No Other Histologic Features, Is Associated With Long-term Outcomes of Patients With Nonalcoholic Fatty Liver Disease	Angulo P	*Gastroenterology*	2015	443	10.1053/j.gastro.2015.04.043
7	The multiple-hit pathogenesis of non-alcoholic fatty liver disease (NAFLD)	Buzzetti E	*Metabolism*	2016	438	10.1053/j.metabol.2015.12.012
8	Nonalcoholic fatty liver disease: a systematic review	Rinella ME	*JAMA*	2015	418	10.1001/jama.2015.5370
9	EASL-EASD-EASO Clinical Practice Guidelines for the management of non-alcoholic fatty liver disease	Marchesini G	*J Hepatol*	2016	380	10.1016/j.jhep.2015.11.004
10	Mechanism of NAFLD development and therapeutic strategies	Friedman SL	*Nat Med*	2018	377	10.1038/s41591-018-0104-9

We found that the highest-ranking cited reference was a meta-analysis review published by *Hepatology* in 2016 (25). The investigators retrieved and analyzed data from 8,515,431 patients from studies published from 1989 to 2015, to determine the prevalence, incidence, risk factors, and long-term outcomes of patients with non-alcohol lipid liver disease (NAFLD). The second-highest-ranked paper was also a review published in *Hepatology* in 2018 (26), which focused on the diagnosis and management of MAFLD. The third-highest-ranked paper discussed the trends, predicted risk factors, and prevention of the global burden (27). These three review articles discussed the pathological and physiological processes of MAFLD clinically and molecularly. The literature cited within papers reflects the authors’ perspectives and research directions. The most highly cited papers listed in **Table 4** have made great contributions to MAFLD research and are the most recognized papers in this field.

In addition, we also analyzed the strong citation burst of references in this field. **Figure 6B** shows the reference of the top 20 articles with the strongest citation bursts. “Begin” refers to the time when the reference was first cited, and “end” refers to the year when the final reference was identified. Seven of the articles are still widely cited. These latter articles have played an important guiding role in the research of MAFLD. Rinella ME published a systematic review in *JAMA* in 2015 (28) with the longest citation duration. The strongest citation reference is an article on MAFLD diagnosis and management published in *Hepatology* by Chalasani N in 2018 (26). Several other references investigated the long-term outcomes of MAFLD development into liver fibrosis (23, 29). MAFLD is a multisystem disease that can increase the risk of type 2 diabetes, cardiovascular disease, and chronic kidney disease (30). Accordingly, the global burden caused by MAFLD is high and has been increasing (25, 27).

**Figure 7:**
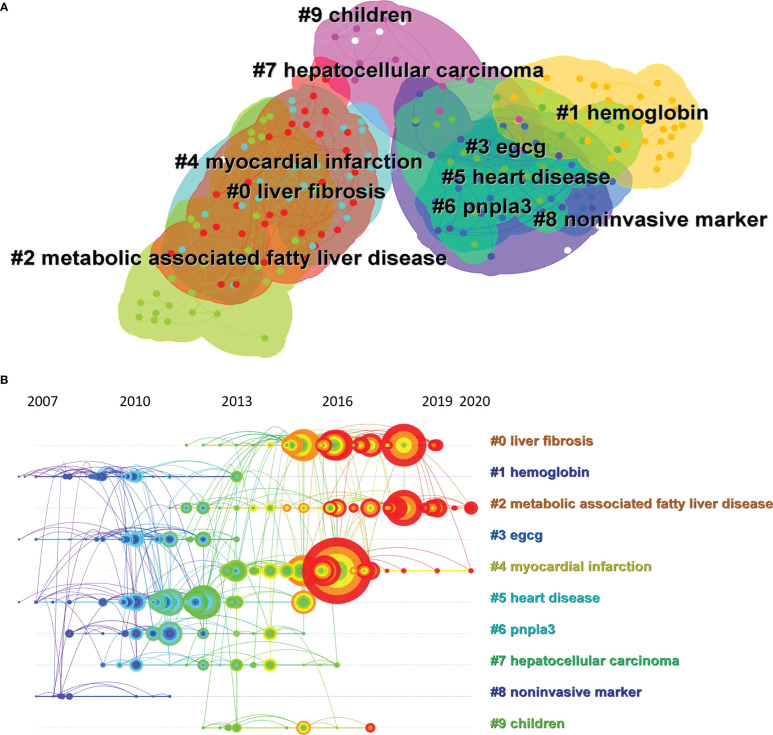
Analysis of co-occurring keywords in MAFLD publication references from 2012 to 2021. **(A)** The cluster network with co-citation was established through CiteSpace. The top 10 clusters of cited articles with MAFLD studies are shown. **(B)** A timeline view of the top 10 largest clusters of citing articles in the field of MAFLD. Right side = cluster labels.

The CiteSpace map of co-citations clustered according to keywords generated from the references of the 13,959 citing articles is shown in **Figure 7A**. The analysis of co-citation clusters revealed that the most relevant terms on MAFLD research by the way of hierarchical cluster labels included #0 liver fibrosis, #1 hemoglobin, #2 metabolic associated fatty liver disease, #3 egcg, #4 myocardial infarction, #5 heart disease, #6 pnpla3, #7 hepatocellular carcinoma, #8 noninvasive marker, and #9 children. The number of cluster tags was inversely correlated with the number of articles that each cluster included. In other words, the cluster marked as #0 contained the largest number of papers among the 228,186 co-cited references. The redder the color patch to which each cluster belongs, the more frequently the references in this cluster were cited in recent years. A summary of clusters is listed in **Table 5**.

**Table 5:** Summary of 10 Clusters.

Cluster ID	Top term	Size	Silhouette*
0	Liver fibrosis	34	0.889
1	Hemoglobin	33	0.725
2	Metabolic associated fatty liver disease	30	0.767
3	egcg	27	0.735
4	Myocardial infarction	24	0.82
5	Heart disease	23	0.924
6	Pnpla3	15	0.984
7	Hepatocellular carcinoma	13	0.828
8	Noninvasive marker	9	0.891
9	children	9	0.777

*Silhouette value >0.5 means the clustering results are reliable.

### 3.7 Analysis of research trend and burst detection with keywords

To clearly describe the shift of hot spots in MAFLD research in the past 10 years, a timeline view is shown in **Figure 7B**. Each circle represents the main cited paper in a cluster and the citation tree-rings of different sizes on the timeline represent the citation rates. We found that the clusters of metabolic associated fatty liver disease starting in 2011 occupied the highest degree of citation bursts until 2020, followed by liver fibrosis. The focus of research in MAFLD shifted from metabolic syndrome to metabolic associated fatty liver disease.

**Figure 8:**
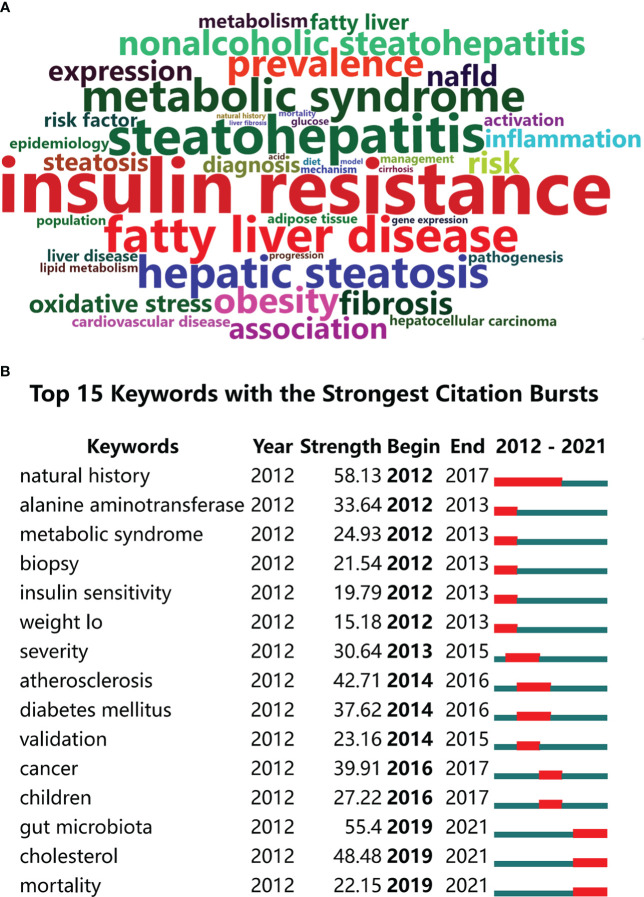
Between 2012 and 2021, keywords analysis and burst detection of publications on MAFLD studies. **(A)** Word cloud analysis of the top 100 high-frequency keywords related to MAFLD research through the bibliometrix R software package. **(B)** Keywords with the strongest burst strength of the 13959 articles on MAFLD research between 2012 and 2021. Keywords marked in red indicate a sudden increase in usage frequency of this keyword during that period. Green represents a relatively unpopular period. The top 15 keywords are shown.

The word cloud shown in **Figure 8A**; it represents the top 100 high-frequency keywords in MAFLD research. Font size is correlated with frequency. After deleting keywords that we identified as holding no practical significance for our research trend analyses, insulin resistance, steatohepatitis, and metabolic syndrome were the highest frequency keywords.

Keyword burst detection is another method that helps to quickly identify research hot spots. **Figure 8B** shows the top 15 references with the strongest keywords bursts on research during the period of 2012-2021. The green line indicates the time range of 2012-2021 and the red line indicates the period that the burst maintains. Amongst the identified keyword bursts, those featured by the end of 2021 were led by gut microbiota with a strength of 55.4, cholesterol with a strength of 48.48, and mortality with a strength of 22.15. The keyword gut microbiota and cholesterol indicated the pathogenesis and therapeutic implications. The investigators’ concern about the mortality related to MAFLD shows that the disease has become a key risk factor for hepatocellular carcinoma. Without effective treatments, MAFLD may become an important cause of future mortality.

## 4 Discussion

In this bibliometric analysis study, we found 13,959 MAFLD research articles published from 2012 to 2021 in the Web of Science database (SCI-E). The overall publication numbers maintained a stable growth trend. Since 2018 there has been a booming growth in publications. The United States showed the greatest international cooperative efforts, while China published the largest number of papers. The representative clusters of cited articles are liver fibrosis and hemoglobin. Gut microbiota, atherosclerosis, and cholesterol were the most intensively utilized keywords over the past decade. This study is the first bibliometric analysis article on MAFLD/NAFLD over the past 10 years and demonstrates a systematic overview of this field and guides future studies. With the help of our bibliometric analysis, researchers interested in MAFLD can obtain a general understanding of the field and quickly grasp the latest research hot spots.

The annual publications output in this field indicates a continuous and steady upward trend in the last 10 years. The increasing rate accelerated from 2018, indicating that the study of MAFLD has gained greater research attention. **Figure 2B** shows that China and the USA were the two leading contributors to MAFLD research. Since 2015, the number of articles published in China has increased annually and surpassed the USA. With rapid life transitions, the increasing burden of MAFLD in China has emerged as a major public health issue. Zhou F et al. found that the National prevalence of MAFLD is 29.2% (31). Due to the increased incidence rate of MAFLD, China has provided greater financial support for MAFLD research. Greater international cooperation is an important trend and is more conducive to the output of high-quality research results. As for the collaborations between countries/regions shown in **Figure 3A**, the USA ranked first in terms of the cooperative relationship with other countries/regions, especially with China. Five of the top 10 institutes ranked by publication output were from China and their contributions to this field were particularly concentrated in recent years (**Figure 3B**). The five responsible Chinese universities are highly regarded for their research acumen. Therefore, these results demonstrate the importance of Chinese efforts and the potential scientific innovation in the MAFLD field. Meanwhile, USA also holds an important position and influence in the MAFLD field. There are many MAFLD patients in the United States. The country shows a good foundation of medical research and has great financial support for research. In general, with the irreversible occurrence of globalization, more cooperation between countries/institutions is still needed for research in this field.

Rohit Loomba from the University of California, contributed the most articles (123), mainly studying treatments and the prognosis of MAFLD. His article published in *Hepatology* studied the therapeutic effect of combination therapies including Cilofexor and Firsocostat on the development of MAFLD (32). His recent research also included the efficacy and safety of the fatty acid synthase inhibitor TVB-2640 in the treatment of MAFLD patients (33) and the effect of aldafermin treatment on gut microbiota (34). Nobili V studied MAFLD with other diseases, such as diabetes, and studied the role of abnormal glucose tolerance as a predictor of liver disease severity (35). Byrne CD called for greater attention to MAFLD (36), which is regarded as a complex metabolic disease, for example, it may be a risk factor for myocardial dysfunction (37).

Amongst the top 10 most cited journals, the journal *Hepatology* had the most citations (11,517). Papers published in *Gastroenterology* had the most average citations per paper (47.37) and likely contributed to its high impact factor (33.883). The journal *PLoS One* had the largest publication count (465 papers) while its average citation per paper was 7.77, which implied that the papers published in *PLoS One* were of less interest and thus resulted in fewer citations. Five of the ten journals were from the USA, showing that the USA provides an important platform for the development of the MAFLD field.

In the analysis of the 228,186 references from 13,959 articles, the top 10 most cited authors are shown in **Figure 6A**; they were all cited over 1000 times. These authors provide an important basic reference direction for MAFLD research. Younssi ZM discussed the impact of renaming NAFLD to MAFLD (38) and compared the long-term outcomes of NAFLD and MAFLD (39). Moreover, Younssi ZM has also made important contributions to MAFLD inspection methods (40), treatment measures (9), MAFLD epidemiology research (41), etc. Kleiner DE summarized the relationship between histological disease activity and the progression of MAFLD (42).

Through the timeline view of MAFLD research hotspot shifts, we found that, in addition to clusters such as liver fibrosis and metabolic fatty liver disease, children and hemoglobin, which seemed to have less correlation with MAFLD, were also highlighted. MAFLD is one of the most common chronic liver diseases in children in the developed world. It is estimated that the worldwide prevalence of pediatric NAFLD is up to 7.6% in the general population and is 34% in the obese pediatric population (43). After 2010, researchers gradually realized that MAFLD not only threatened the health of adults, but also could not be ignored in children (44). Therefore, researchers began to pay more attention to the development and treatment of MAFLD in children. However, compared with MAFLD research for adults, the attention paid to children’s MALFD is not very high. From 2016 to 2018, scholars were very concerned about MAFLD among children (45–47), which is shown as red node in **Figure 6B**. But in recent years, researchers have paid less attention to MAFLD in children. Hemoglobin was highlighted before 2013 as shown in **Figure 6B**. During this period, researchers focused on hemoglobin research because they found that many MAFLD patients had diabetes (48, 49), and glycosylated hemoglobin was an important indicator of diabetes (50). Therefore, it was shown as hemoglobin in the graph by clustering. However, hemoglobin is not a key factor in the occurrence of MAFLD. Therefore, few studies have focused on the role of hemoglobin in MAFLD since 2013.

Keyword burst refers to the keywords that are significantly cited by papers over a specific period; it is considered another important indicator of study hotspots or emerging trends over time. As is shown in **Figure 8B**, the top 15 keyword bursts with the strongest citations are listed, revealing potential hotspots in MAFLD research over the last 10 years. It is worth noting that most of the listed keyword outbreaks began in 2012 and ended before 2021. These changes indicate the fluidity of main research interests over time; in short, research identified by certain keywords formed the MAFLD hotspot at a specific period but these keywords did not necessarily persist into the current research hotspots. Amongst them, the “gut microbiota” keyword burst began in 2019 and lasted until the end of 2021, ranking first with a strength of 55.4. The literature related to this keyword mostly studied the pathogenesis and treatment of MAFLD (51–55). In addition to the discovery of new druggable targets and pharmacotherapeutics, personalized medication, and combinatorial therapies targeting multiple profibrotic pathways could be promising in achieving successful antifibrotic interventions in patients with MAFLD/NAFLD (55–57). It has been shown that the grade of liver fibrosis is related to the prognosis of MAFLD. Liver fibrosis is the most important liver biopsy feature associated with increased overall- and liver-related complications (23). In addition, another study showed that the liver fibrosis stage is the most useful marker to predict future mortality in patients with MAFLD/NAFLD (29). Many studies explore the links between MAFLD/NAFLD, metabolic syndrome, dysbiosis, poor diet, and gut health. Dysbiosis increases gut permeability to bacterial products and increases hepatic exposure to injurious substances that increase hepatic inflammation and fibrosis. Changes to the microbiome can also cause dysmotility, gut inflammation, and other immunological changes in the gut that can contribute to liver injury (58). Juliette et al. found that microbiota-driven gut vascular barrier disruption is a prerequisite for non-alcoholic steatohepatitis development. They also showed that the FXR agonist obeticholic acid can be utilized as a pharmacologic treatment (59).

Secondly, the “cholesterol” keyword emerged in 2019, with a strength of 48.48, continuing until the end of 2021. It has been pointed out that hepatic cholesterol transport plays an important role in MAFLD and atherosclerosis (60) and cholesterol plays a significant role in the transition of MAFLD to NASH (61). Cholesterol is a significant risk factor for non-alcoholic steatohepatitis. High dietary cholesterol led to the sequential progression of steatosis, steatohepatitis, fibrosis, and eventually HCC in mice. Cholesterol-induced MAFLD-HCC formation was associated with gut microbiota dysbiosis. The microbiota composition clustered distinctly along stages of steatosis, steatohepatitis, and HCC. Moreover, atorvastatin restored cholesterol-induced gut microbiota dysbiosis and completely prevented MAFLD-HCC development (12).

The third keyword burst that lasted until the end of 2021 was “mortality”. The concern about MAFLD mortality indicates that the disease has become a public problem threatening health and life (62, 63). These keywords indicated that MAFLD is a metabolic disease stemming from multiple causes, and that there is no clear and effective clinical treatment. Overall, these results are inseparable from the occurrence and development of MAFLD and reflect an impact on future hotspots, leading the way for subsequent research.

Our study has some limitations. Firstly, the data analyzed were only extracted from the SCI-E database from WoSCC, and records from other important search engines such as PubMed, Embase, and Ovid were excluded, potentially resulting in an incomplete representation of the publications on MAFLD/NAFLD over the past 10 years. However, data retrieved from WoSCC included comprehensive records such as title, author, institute, and reference which are necessary for bibliometric analysis. In addition, only data retrieved from WoSCC contains complete information for co-citation analysis by CiteSpace whereas other databases do not. Secondly, English is still the preferred language for academic journals today, therefore we focused on papers published in English, which resulted in an omission of articles published in other languages.

## 5 Conclusion

With the help of bibliometric mapping, we analyzed the research on MAFLD in the past 10 years. Since 2018, the number of publications has increased rapidly. The United States and China made the largest contributions to the MAFLD field. The United States was in the leading position in terms of international cooperation, while China has led in the number of publications since 2015. The current research mainly focuses on liver fibrosis and gut microbiota. Gut microbiota may be an important research direction to investigate the transition of fatty liver to liver fibrosis. This study guides future research work, which may help to discover the mechanism of MAFLD and new treatment methods.

## Data availability statement

The raw data supporting the conclusions of this article will be made available by the authors, without undue reservation.

## Author contributions

PG and WY put forward the concept of this study and designed this study. YL and LW screened articles and wrote original manuscript. YL, LW, and FL conducted CiteSpace and VOSviewer analysis. PG, WY, YL, LW, FL, LY, YJ, YZ, ZZ, DT, and SX revised and critically edited the manuscript. All authors contributed to the article and approved the submitted version.
