# Supplementary material for: Emerging trends and hotspots in metabolic dysfunction-associated fatty liver disease (MAFLD) research from 2012 to 2021: A bibliometric analysis
Source: Front Endocrinol (Lausanne). 2023 Jan 24;14:1078149. doi: 10.3389/fendo.2023.1078149 (PMC9904363; doi:10.3389/fendo.2023.1078149)
Supplement: Supplementary file 1 [file DataSheet_1.docx]

Supplementary Material


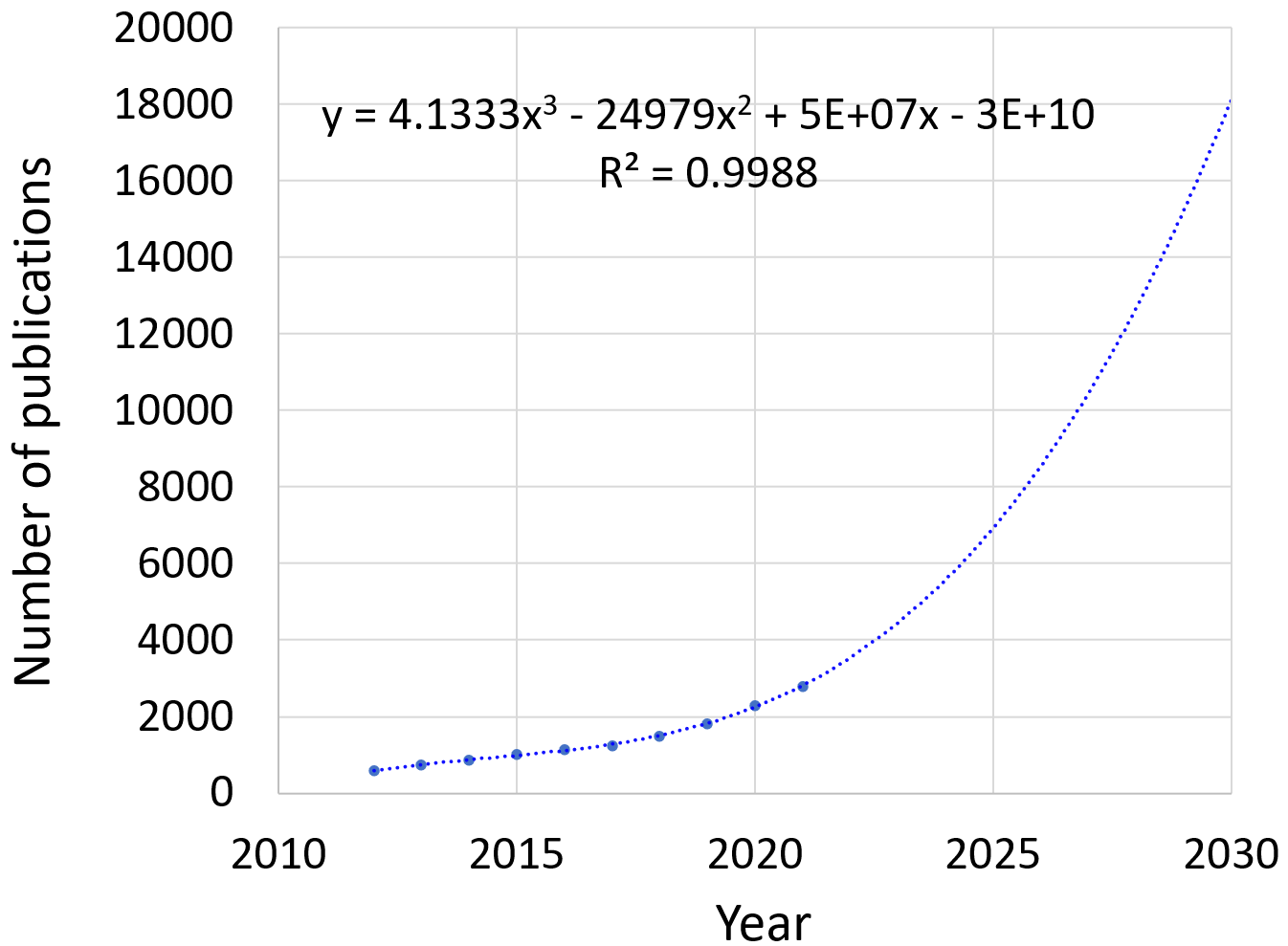


**Supplementary Figure 1**. Output of publications and growth prediction of MAFLD research. The number of publications from 2012 to 2021 are presented by scatters. The dashed line represents the predicted curve, R^2^=0.9988.
